# Psychometric and next day electrodermal activity data from an experiment involving midazolam, ketamine, and painful stimulation

**DOI:** 10.1016/j.dib.2023.109634

**Published:** 2023-10-01

**Authors:** Lindsay Zhang, Keith Vogt

**Affiliations:** Department of Anesthesiology and Perioperative Medicine, University of Pittsburgh School of Medicine, A-1305 Scaife Hall, 3550 Terrace Street, Pittsburgh, PA 15261, USA

**Keywords:** Galvanic skin response, Pain, Anxiety, Conditioning

## Abstract

This is a report of unpublished data obtained from a randomized, single-blind, within-subject crossover design, neuroimaging trial comparing the effects of midazolam and ketamine on memory and pain. An experimental auditory paradigm paired with the periodic pain was used to examine auditory encoding and subsequent recognition. Psychometric surveys assessed stress, anxiety, depression, and pain behaviors. These data were collected to potentially characterize inter-subject variation during analysis. Electrodermal activity was measured to examine the association between previous pain-pairing of experimental items and autonomic responses during next day recognition memory testing, with exposure to the same auditory word items. Electrodermal responses were determined using an event-related analysis approach. These data may help shape future experiments using psychometric data to characterize individual responses or using next day electrodermal activity to determine conditioned responses to previously-experienced aversive stimuli.

Specifications Table

**Table:** 

Subject	Neuroscience: Behavioral
Specific subject area	Memory and conditioning following minimal sedation with midazolam and ketamine
Data format	Raw
Type of data	Table
Data collection	Subject-reported psychometric data were obtained using the following written questionnaires: the Brief Inventory of Perceived Stress, the Beck Depression Inventory, the Pain Anxiety Symptom Scale-Short Form 20, Pain Vigilance and Awareness Questionnaire, the Pain Catastrophizing Scale, and the State-Trait Inventory for Cognitive and Somatic Anxiety. Electrodermal activity was acquired using a BIOPAC MP160 analog-to-digital conversion system and captured using the accompanying software, AcqKnowledge version 5.0. EL507 electrodes and BIOPAC isotonic electrode gel were used. If there were less than 10 event-related responses that exceeded a threshold of 0.04 μS, then the subject's electrodermal activity data were excluded.
Data source location	Institution: University of Pittsburgh City/Town/Region: Pittsburgh, Pennsylvania Country: United States of America Latitude and for collected samples/data: 40.440128, -79.961288
Data accessibility	Repository name: Open Science Framework Data identification number: 10.17605/OSF.IO/YNPQG Direct URL to data: https://osf.io/ynpqg/
Related research article	K.M. Vogt, J.W. Ibinson, C.T. Smith, A.T. Citro, C.M. Norton, H.T. Karim, V. Popov, A. Mahajan, H.J. Aizenstein, L.M. Reder, J.A. Fiez, Midazolam and ketamine produce distinct neural changes in memory, pain, and fear networks during pain, Anesthesiology. 135 (2021) 69–82. 10.1097/ALN.0000000000003774.

## 1 Value of the Data

• Researchers may apply the presented psychometric data in discerning how drug exposure influences inter-subject variation in mood and pain behaviors.
• The presented electrodermal activity data demonstrate the relationship between prior painful stimulation paired with non-painful auditory stimuli and are useful to demonstrate conditions under which conditioned responses may be revealed on re-exposure to the non-painful stimuli.
• Researchers investigating memory formation, in the context of periodic painful stimulation, and hoping to develop an aversive conditioning paradigm may benefit from these data.
• These data may help shape future experimental designs seeking to use psychometric data to characterize individual responses or using next day electrodermal activity as a measure of conditioned responses in similar paradigms that employ aversive stimuli.

## 2 Data Description

**Fig. 1:**
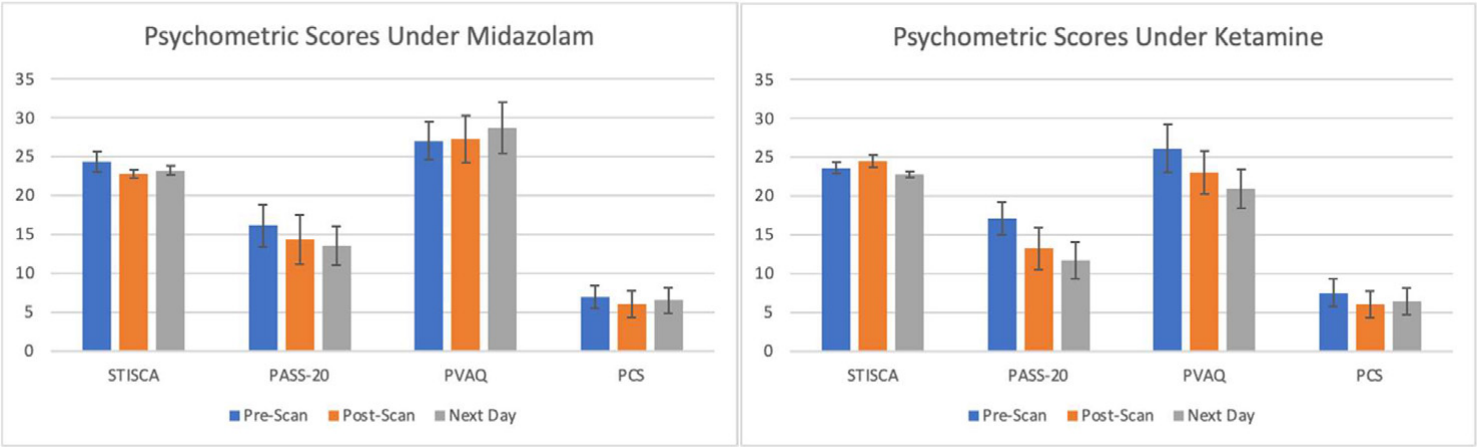
Average scores for STICSA, PASS-20, PVAQ, and PCS prior to the scan, after the scan, and the next day, under midazolam and ketamine. Error bars represent standard error.

Psychometric data were collected from 25 healthy subjects at three time points: pre-scan, post-scan, and during the next day follow-up visit, in which there was no MRI scanning. Psychometric data is available except for missing pre-scan (1), post-scan (15) and next day follow-up (13) scores due to this data not being collected at these time points in the first few sessions. Tabulated final total scores are presented for each of the following questionnaires: the Brief Inventory of Perceived Stress (BIPS) [1], the Beck Depression Inventory (BDI-2) [2], the Pain Anxiety Symptom Scale-Short Form 20 (PASS-20) [3], Pain Vigilance and Awareness Questionnaire (PVAQ) [4], the Pain Catastrophizing Scale (PCS) [5], and the State-Trait Inventory for Cognitive and Somatic Anxiety (STICSA) [6] in an Excel table. In Fig. 1, average scores are provided for psychometric inventories completed during the beginning and conclusion of memory encoding sessions and at the beginning of next day memory testing sessions.

Electrodermal activity data (EDA) refers to changes in the electrical properties of the skin caused by sweat secretion. It was collected during the next day sessions are provided in the supplementary materials as comma-separated value (txt) files for each subject. The file names are labeled by subject and session number (e.g., MMA_SubjXX_SesX_RKN.txt). A header is displayed at the top of the text file indicating the subject and session number (as part of the file name); this is followed by the sampling rate, and then the labels and units for each acquisition channel. Five channels were recorded: CH2, CH4, CH5, CH6, and CH9. Column 1 denoted by the “min” header, represents the time (in minutes) for each sample. Column 2, titled CH2, contains the values of the raw EDA amplitudes in microsiemens. Column 3 or CH4 contains signal amplitude values in volts, corresponding to a marker for the timing of previously-heard words that were previously paired with pain. CH5 indicates timing for previously-heard non-pain words. CH6 marks the timing of new words (not heard the previous day). CH9 presents the raw amplitude values from tracing of the electrocardiograph, in millivolts.

**Fig. 2:**
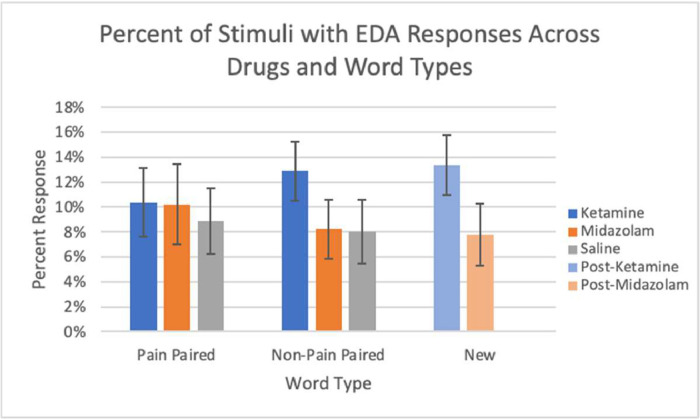
The percent of EDA responses following a stimulus across all drug conditions and word types: pain paired, non-pain paired, and new. Error bars represent standard error.

**Fig. 3:**
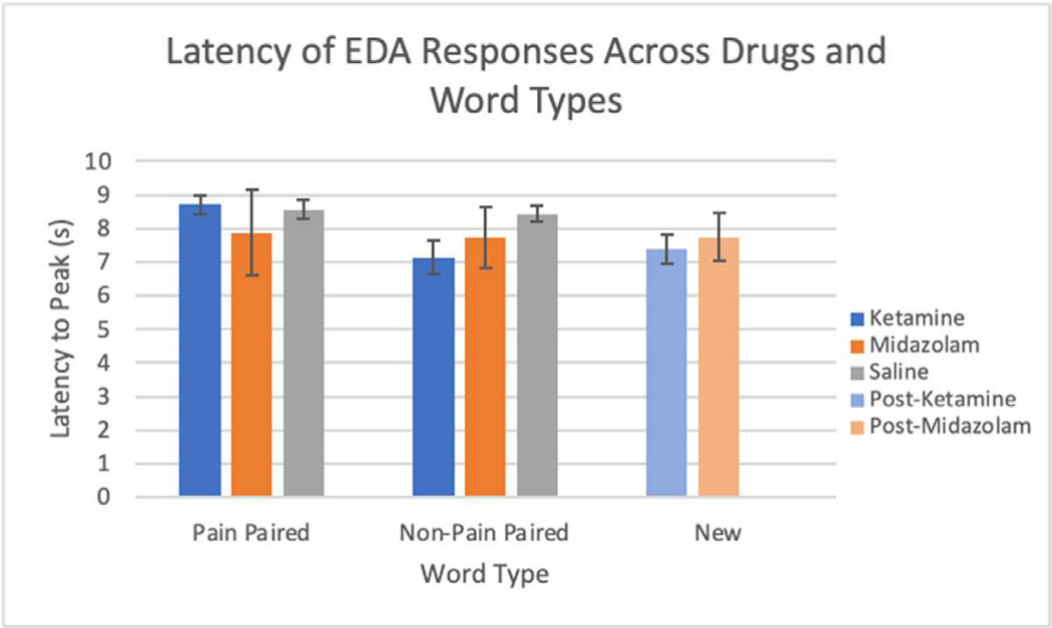
The latency to peak in seconds (s) of EDA responses following a stimulus across all drug conditions and word types: pain paired, non-pain paired, and new. Error bars represent standard error.

**Fig. 4:**
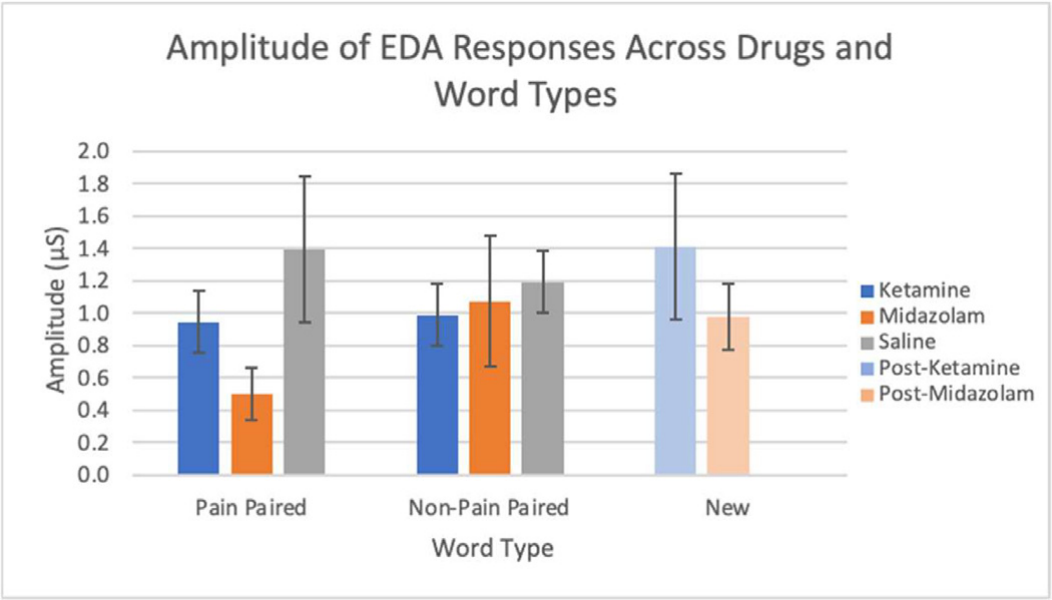
The amplitude in microsiemens (μS) of EDA responses following a stimulus across all drug conditions and word types: pain paired, non-pain paired, and new. Error bars represent standard error.

EDA data from the next day (post-scan/drug) sessions are available from 23 subjects. Missing data were due to technical issues, most commonly a lack of capturing skin responses. EDA was analyzed by the percent of EDA responses, latency, and amplitude across stimulus types with pain associations and drug conditions. The presented stimuli from the memory testing sessions are classified into three different word types: pain paired (words presented with a shock during the memory encoding sessions), non-pain paired (words not associated with a shock during memory encoding sessions), and new (words not heard during the memory encoding session). The percent of EDA responses from both midazolam and ketamine conditions are shown, according to word type (Fig. 2). The latency of EDA responses, determined by time to peak in seconds, for each word type is shown in (Fig. 3). The amplitudes of the EDA responses, in microsiemens, are depicted in (Fig. 4) by word type. New words appeared only at next day testing, so are shared between the saline and drug conditions from the previous day's encoding session.

## 3 Experimental Design, Materials and Methods

### 3.1 Experimental Design & Methods

Subjects first completed six psychometric questionnaires (BIPS, BDI-2, PASS-20, PVAQ, PCS, and STISCA) outside the scanner environment. Questionnaires were administered by research staff. This was done prior to IV start and any painful stimulation. Using a randomized, single-blind, within-subject crossover design, subjects performed the experimental paradigm in an MRI scanner (details provided previously [7]) under saline followed by one of the drugs. This sequence was repeated in a subsequent session with the other agent (i.e. different drug on different days).

Light sedation was achieved with both drugs, with subjects able to respond to verbal commands. During the experiment, subjects periodically experienced painful electrical stimulation (rated 7/10, based on previous titration) delivered to their left index finger from an electric nerve stimulator (EzStim II; Life Tech, USA). They made categorization decisions about a series of words they were hearing; one-third of the words were paired with pain. All these words were repeated three times, in randomized order, with pain-pairing kept consistent. Following the scan, the STICSA, PASS-20, PVAQ, and PCS questionnaires were completed again.

The following day, ranging 20-32 hours after the memory encoding sessions, subjects returned for a follow-up testing session. The STICSA, PASS-20, PVAQ, and PCS assessments were again completed at the beginning of the visit. EDA electrodes were placed on the left palm to detect evidence of remembered physiological responses from previously pain paired words. No painful shocks were administered during these next day follow-up sessions. Subjects performed a Remember-Know recognition paradigm [8]; while listening to a series of words, subjects were tasked with identifying which were previously-heard the prior day in the scanner.  All words that were previously-heard were included, along with an equal number of new (not previously-heard) word items, in a randomized order.

### 3.2 Analysis

EDA data were processed within the AcqKnowledge software. Electrodermal response peaks occurring following the onset of the word item stimuli were considered paired together if the following parameters were met, similar to our previous report [9]. Electrodermal response peaks less than 10% of the maximum amplitude were rejected, and an absolute threshold of 0.04 μS change was used to detect an EDA response. A high pass filter of 0.05 Hz was used in addition to a baseline estimation window width of one second. To be considered valid, EDA responses had to fall more than 0.3 s and less than 11 s after the corresponding word stimulus onset.

### 3.3 Materials

Subjects completed psychometric questionnaires to capture the subjective experience of pain and associated subject-reported measures. The BIPS [1] measures perceived stress by the number of incidents in the past month where subjects felt like they “Had too many things to do,” or “Felt under pressure from deadlines.” The BDI-2 [2], measures the severity of depression symptoms in groups of statements. Using a scale of 0–3, subjects rate the extent of behaviors such as past failure (0 I do not feel like a failure; 1 I have failed more than I should have; 2 As I look back, I see a lot of failures; 3 I feel I am a total failure as a person) or crying (0 I do not cry anymore than I used to; 1 I cry more than I used to; 2 I cry over every little thing; 3 I feel like crying, but I cannot). The PASS-20 [3] measures pain-related anxiety and fear. This questionnaire rates items such as “I cannot think straight when in pain,” or “I worry when I am in pain,” in terms of frequency. The PVAQ [4] contains two reverse scored items. This questionnaire indicates frequency of behaviors such as “I am very sensitive to pain,” and “I become preoccupied with pain,” that were experienced in the past two weeks. The PCS [5] is used to quantify subjects’ pain experiences. Items assess subjects’ agreement with statements beginning with “When I am in pain...” and ending with phrases such as “I feel I cannot go on,” and “I feel I cannot stand it anymore.” The STISCA [6] assesses current physical and mental symptoms of anxiety with statements such as “My heart beats fast,” or “My muscles are tense.” Each psychometric questionnaire was scored by summing the responses to all items, with higher scores indicating increased effect being measured. Details regarding the scoring of items are given in Table 1.

**Table 1:** Psychometric questionnaire item scoring and total score ranges.

Questionnaire	Number of Items	Scale	Total Score Range
BIPS [1]	9	0 never; 4 very often	0-36
BDI-2 [2]	21	0;3	0-63
PASS-20 [3]	20	0 never; 5 always	0-100
PVAQ [4]	16	0 never; 5 always	0-80
PCS [5]	13	0 not at all; 4 all the time	0-42
STISCA [6]	21	1 not at all; 4 very much	21-84

During the follow-up testing portion of the experiment that took place the next day, subjects were seated in front of a laptop computer and heard experimental word items through headphones while using keyboard (number pad) buttons to respond. This session did not take place in an MRI scanner. EDA data were collected using EL507 electrodes loaded with BIOPAC isotonic electrode gel for EDA (GEL101A). EDA acquisition was done with a BIOPAC MP160 (BIOPAC Systems, Goleta, CA) analog to digital conversion system and captured using the accompanying software, AcqKnowledge version 5.0 (BIOPAC Systems, Goleta, CA). The data sampling rate was 625 Hz.

### 3.4 Subjects

Healthy adults between the ages of 18 and 38 years were recruited from the Pittsburgh community and completed the study. Subjects were compensated $200 for their participation. All subjects reported having no significant memory impairments, significant hearing impairments, chronic pain, other chronic medical issues, and recent or regular use of antidepressants, antipsychotics, antihistamines, anxiolytics, stimulants, sleep aids, and analgesics. Data was collected from a total of 25 subjects (14 male, 11 female) of which the average age was 25.4 years and the age range was 19–37 years. Age and sex for each subject are listed in Table 1 of the original paper [7], in which Subject 2 is Subject 32 in this investigation and the subject numbers following Subject 2 align with the subject numbers following subject 32 in our study.

## Limitations

None.

## Ethics Statement

The experimental protocol was approved by the University of Pittsburgh Institutional Review Board (PRO14050609) and informed consent was obtained from each subject. This study otherwise conformed to the highest standards for the responsible conduct of research, including compliance with the Declaration of Helsinki.

## CRediT authorship contribution statement

**Lindsay Zhang:** Formal analysis, Writing – original draft, Visualization. **Keith Vogt:** Conceptualization, Methodology, Validation, Investigation, Resources, Writing – review & editing, Supervision, Project administration, Funding acquisition.

## Data Availability

Psychometric and next day electrodermal activity data from an experiment involving midazolam, ketamine, and painful stimulation (Original data) (Open Science Framework).
